# Inherent and Composite Hydrogels as Promising Materials to Limit Antimicrobial Resistance

**DOI:** 10.3390/gels8020070

**Published:** 2022-01-20

**Authors:** Rahela Carpa, Alexei Remizovschi, Carla Andreea Culda, Anca Livia Butiuc-Keul

**Affiliations:** 1 Molecular Biology and Biotechnology Department, Faculty of Biology and Geology, Babeş-Bolyai University, 1 M. Kogalniceanu Street, 400084 Cluj-Napoca, Romania; rahela.carpa@ubbcluj.ro (R.C.); anca.keul@ubbcluj.ro (A.L.B.-K.); 2 Center of Systems Biology, Biodiversity and Bioresources, Babeş-Bolyai University, 5-7 Clinicilor Street, 400006 Cluj-Napoca, Romania; 3 Parasitology and Parasitic Diseases Department, University of Agricultural Sciences and Veterinary Medicine, 3-5 Calea Manastur Street, 400372 Cluj-Napoca, Romania; carla-andreea.culda@usamvcluj.ro

**Keywords:** antimicrobial activity, carriers, composites, gene delivery, nanoparticles

## Abstract

Antibiotic resistance has increased significantly in the recent years, and has become a global problem for human health and the environment. As a result, several technologies for the controlling of health-care associated infections have been developed over the years. Thus, the most recent findings in hydrogel fabrication, particularly antimicrobial hydrogels, could offer valuable solutions for these biomedical challenges. In this review, we discuss the most promising strategies in the development of antimicrobial hydrogels and the application of hydrogels in the treatment of microbial infections. The latest advances in the development of inherently and composite antimicrobial hydrogels will be discussed, as well as hydrogels as carriers of antimicrobials, with a focus on antibiotics, metal nanoparticles, antimicrobial peptides, and biological extracts. The emergence of CRISR-Cas9 technology for removing the antimicrobial resistance has led the necessity of new and performant carriers for delivery of the CRISPR-Cas9 system. Different delivery systems, such as composite hydrogels and many types of nanoparticles, attracted a great deal of attention and will be also discussed in this review.

## 1. Introduction

Antimicrobial agents—such as antibiotics—have dramatically reduced the number of deaths from infectious diseases over time; however, they are often overused and discarded in the environment. However, the selective pressure exerted by the use—appropriate and/or inappropriate—of antibiotics has led to the emergence of antibiotic resistance (ABR) [1]. Several bacteria have developed resistance to one or more antibiotics from three or more antibiotic classes, and those resistant to all antibacterial drugs are identified as multi-drug resistance (MDR) bacteria [2]. Even though antimicrobial resistance (AMR) is a major and growing public health concern, the research relating to the growth of this phenomenon in environmental settings is remarkably limited. A key strategic objective of the European Antimicrobial Resistance Surveillance Network, together with the WHO, is to strengthen AMR surveillance, but they recognize that several countries in the region do not have systems for the surveillance of AMR, antibiotic use, and hospital-acquired infections. Multi-drug resistance among the bacterial pathogens is of particular concern because they are responsible for many severe infections in hospitals, as well as the contamination of implants or devices introduced into the body as stents or catheters. Several reports have confirmed a rapid increase in rates of infections due to methicillin-resistant *S. aureus* (MRSA) [3], extended-spectrum beta-lactamase (ESBL) [4] and carbapenemase-producing *K. pneumonia* [5], metallo-beta-lactamase-producing *A. baumannii* [6], metallo-beta-lactamase-producing *P. aeruginosa* (MBL-PA) [7], and extended-spectrum beta-lactamase (ESBL) producing *Enterobacter* spp., *Clostridium dificile, Escherichia coli*, and *Klebsiella oxytoca* [8]. Bacterial pathogens are able to avoid the activity of antibiotic used in medicine [9] due to the numerous different mechanisms: (i) inactivation or alteration of the antimicrobial molecule, (ii) bacterial target site modifications, (iii) reduced antibiotic penetration/accumulation, and (iv) the formation of bacterial biofilms [10]. In addition, because of their ability to form biofilm on biological surfaces, bacterial pathogens are highly prevalent in clinical settings, making it difficult to treat infectious diseases [11]. Although bacteria can be intrinsically resistant to certain antibiotics, they may also accumulate AMR genes from mobile genetic elements (MGE). Thus, the most phenotypic variability in AMR is due to MGEs [12]. As a result, the bacterial genome may contain hundreds of gene sequences that reveal the previous exposure to the foreign DNA. Due to the high pressure exerted by antimicrobial resistance and the increasing prevalence of multiresistant bacteria, the development of new and beneficial treatments is required. Several treatments against multiresistant pathogens have been developed over time, including: new drugs, phage therapy (including derivates), antivirulence therapy, lysins, antibodies, probiotics, and immune stimulation [13]. The particular problem with antimicrobial treatment is the delivery method of the antimicrobial agents and avoiding the systemic uptake that increases the selection of resistant bacteria. Cross-linked polymers (hydrogels) play a fundamental role in the treatment of infectious diseases due to their compatibility with tissues and the loading capacity of different antimicrobials. Hydrogels can serve as antimicrobials, as well as chitosane and other naturally derived polysaccharide [14]. They can also serve as drug delivery systems for antibiotics, metal nanoparticles [15], antimicrobial peptides [16] biological extracts [17], implant coating to prevent infection [18,19] and carriers for delivery of the CRISPR-Cas9 system for curing the plasmid encoding antimicrobial resistance [20,21]. In recent years, the design of coatings suitable for localized treatment of surface-related infections of medical devices was of great interest. Thus, several composite hydrogels were developed to prevent the adhesion of bacteria to the surfaces of medical devices by the inhibition of quorum sensing and biofilm formation [22]. Smart hydrogels targeting bacterial infections and responsive to the bacterial microenvironment, their ability to adjust the release of antibiotics and/or antimicrobial compounds according to the bacterial contamination, have been studied. These strategies limit the accumulation of drugs in healthy host tissues, minimizing the risks of toxicity and the selection of resistant bacteria [23]. A lower dose of antibacterial compounds could be administered with hydrogels than when administered systemically, thus overcoming the bacterial resistance [24]. Due to the multiple mechanisms of antibacterial ingredients loaded in hydrogels, it is difficult for bacteria to develop resistance. Moreover, different ingredients might exhibit a synergic effect, increasing the antibacterial spectrum and antimicrobial effect. As hydrogels have offered a new way to fight against resistant bacteria, numerous studies and clinical applications are produced yearly.

**Figure 1:**
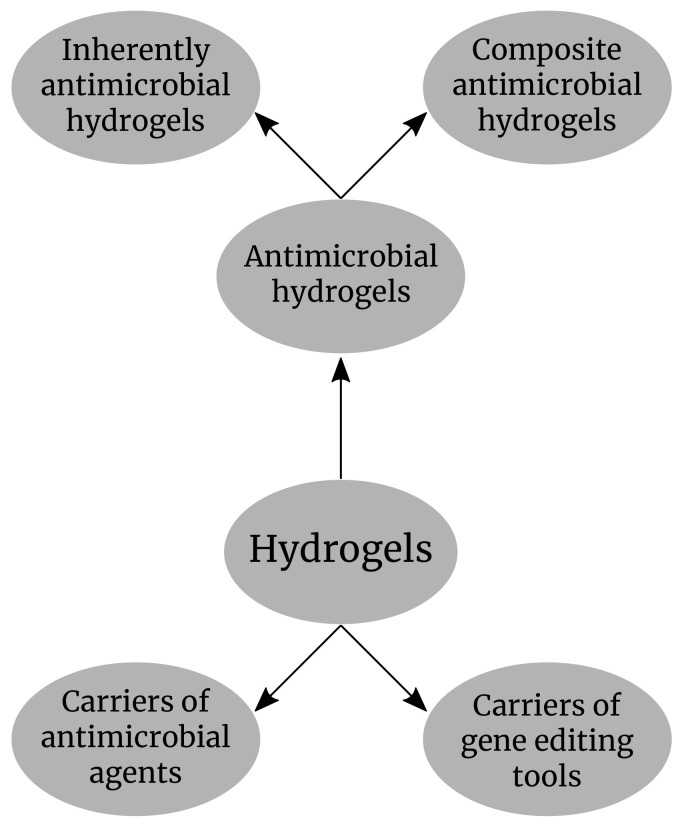
Hydrogels as antimicrobial compounds and carriers of antimicrobial agents and gene editing tools.

In this review, we discuss the latest advances in the development of different types of antimicrobial hydrogels and the main application in limiting the antimicrobial resistance and the potential application of the hydrogels in gene delivery during the curing of bacterial plasmids containing the resistance genes with the CRISR-Cas9 technology. The main application of hydrogels in antimicrobial resistance that will be discussed in this review is shown in (Figure 1).

## 2. Classification of Hydrogels

**Figure 2:**
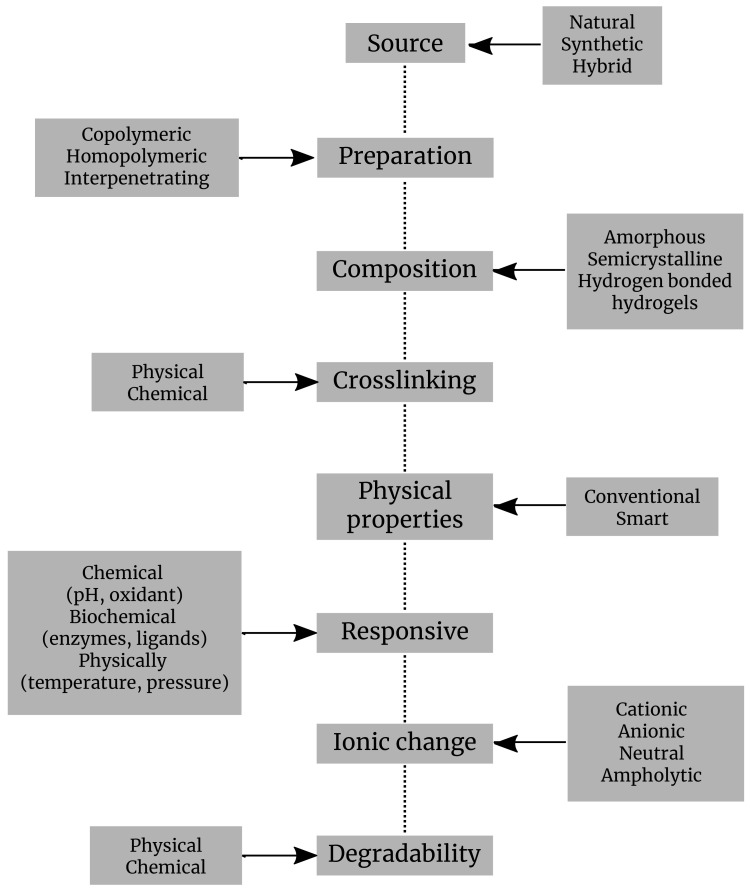
Classification of hydrogels by different criteria.

Hydrogels are classified based on various criteria: sources, preparation, composition, cross-linking, physical properties, ionic charge, degradability, responsive, administration, etc. (Figure 2).

- Depending upon their origin, hydrogels can be split into natural, synthetic, or hybrid hydrogels. Generally, hydrogels with natural origin exhibit a superior biocompatibility and favor biological processes, while the synthetic hydrogels exhibit more consistent mechanical and biochemical attributes. The hydrogels of natural origin are obtained based on precursors belonging to different structural categories of biopolymers, chains representing polysaccharides, or peptides/proteins [25];
- Depending upon their preparation, hydrogels were defined in various ways. The most popular definition which describes a hydrogel as a cross-linked polymeric network which is water-swollen, derived from the basic reaction of one or more units of monomer/polymer/cross-linker. A different description presents it as a polymeric material capable of swelling and retaining a large amount of water in its three-dimensional matrix, but which does not dissolve in water [26]. They are also illustrated as polymeric systems that present the capability to swell in water and retain a significant proportion of water inside their three-dimensional net, without dissolving in water. Food and biomaterial researchers are using two similar terms, gels and hydrogels, to describe polymeric cross-linked net structures [27]. Homopolymers are the polymers which have only one type of monomer in their assemblage. They may have a cross-linked structure, due to the nature of the monomer and the technique of polymerization. Copolymeric hydrogels are the ones that are made of two types of monomers, at least one of them being hydrophilic [28];
- Hydrogels can be also classified according to their **structure**, which may be amorphous, semicrystalline, crystalline, or hydrocolloid [28];
- As the hydrogels are basically built by **cross-linking** networking, therefore based on cross-linking, they are classified regarding this feature into two categories: (a) physically cross-linked or self-assembled hydrogels are formed through reversible bonds based on ionic interactions, crystallization, formation of stereocomplex, hydrophobization of polysaccharides, interaction of proteins or hydrogen bonds; (b) a chemically cross-linked hydrogel, linked by permanent covalent bonds which can be polymerized by chain growth, addition, and condensation [27]. Several types of physical and chemical hydrogels were prepared from natural or synthetic polymers in order to be used in miscellaneous applications (Table 1).
- As regards the administration to patients, hydrogels are either implanted or injected. Injectable hydrogels are preformed before injection or are formed in situ [41];
- Depending upon their response, the hydrogels are broken down into physically, chemically, and biochemically responsive hydrogels. They can further be designed to be responsive to environmental variables, such as temperature, light, pH, antigens, or even enzymes. Hence, hydrogels can be divided into physical, chemical, or biochemical classifications. Physical hydrogels can pass from liquid to gel in response to a specific change in environmental parameters, such as temperature, pH, concentration of ions, or changes in the state of two such components. Chemical gels use covalent bonding that provides mechanical integrity and degradation resistance in comparison with other weak materials. In biochemical hydrogels, the gelation process is performed with the involvement of biological agents, as enzymes or amino acids [14];
- According to their ionic charge, hydrogels can be designated as cationic, anionic, neutral, and ampholytic. For instance, poly(norbornene) is a cationic polymer, and it was thoroughly scrutinized for its antimicrobial properties [42];
- Depending upon their physical properties, there are two types of hydrogels: conventional and smart hydrogels. The first are the ones already known, previously established in the past. Smart hydrogel systems include elements capable of chemically or structurally displaying responses to a range of external stimuli comprising light, temperature, concentration of ions, pH, chemicals, and even magnetic or electric fields. This change in structure and volume as a response to the stimuli as the ones above opens a huge research potential and a large array of applications [43];
- Depending upon their degradability process, the hydrogels are split into two types: biodegradable and non-biodegradable. The biodegradability and biocompatibility make them a strong candidate for biological and environmental applications, as implants or materials for pollutants removal. They can even bring biodegradability to electronics, meaning that hydrogels represent a new option for the designing and creation of supercapacitors. Natural hydrogels are not only biodegradable and biocompatible. For instance, chitosan has become the preferred hydrogel for developing antimicrobial hydrogels of natural origins, as its properties include fast cross-linking [14]. Hydrogels can be engineered to fit a number of large range application due to their pliability, the possibility to be modulated according to needs [14].

## 3. Processing Procedure

Polysaccharides-based natural hydrogels can be synthesized by different methods, which can be either chemical or physical. Chemical methods are based on the cross-linking of the component parts already existing in the gelation feed mixture. Hydrogels are physically obtained, from polysaccharides, by the freeze–thaw technique. The polysaccharide hydrogels resulting from this technique exhibit more desirable characteristics than the conventional ones, which result from cross-linking. The physically obtained hydrogels are tightened by multiple inter-chain hydrogen bonds, within the polymeric structure. The freeze–thaw technique also allows, by modulating parameters such as temperature, freezing span, polysaccharides types, kinds of soluble additives, and number of refreezing cycles, to regulate the properties of the final product. A large array of polysaccharides, including, for example, the hyaluronic acid, carboxymethylated cellulose, carboxymethylated-curdlan, locust bean gum, xanthan, b-glucan, starch, agarose, and maltodextrins, can be involved in obtaining gels [27]. Polysaccharide polymers are preferred, instead of synthetic polymers, for the forming of many hydrogels, due to their traits of biocompatibility, biodegradation, or good hydrophilicity. Hence, they are used in biomedical, industrial, and even environmental applications. There are several ways to obtain synthetic hydrogels. Chemically cross-linked hydrogels look like 3D polymeric structures held by plentiful of bonds between the chains. Many of them are connected by covalent bonds which can form directly between hydrophobic monomers. Commonly involved in such monomers are vinyl pyrrolidone, methacrylic acid, and poly-2-hydroxyethyl methacrylate. A very suitable and often used method for cross-linking is applying the use of hydrolysis/radiation for the hydrophobic polymeric network [44]. The physical interactions required to develop hydrogels include crystalline junctions, hydrogen bonding, and phase-separation. The strength of the obtained hydrogels is directly related to the strength and density of the physical bonds. However, despite being relatively weak, the hydrogen bonds can still hold a stable structure. They lead to crystalline junction points in the chain of the polymer that translate within hydrogels [44]. Poly-vinyl alcohol is synthetic and water soluble, and several mechanisms can be used to transform this polymer into hydrogels. For example, covalent cross-linking or hydrogen bonds can be involved to develop a hydrogel derivative hydrogels based on poly-vinyl alcohol by the repeated freeze–thaw technique [27].

## 4. Inherently Antimicrobial Hydrogels

### 4.1. Natural Hydrogels

Antimicrobial hydrogels, based on natural polymers, can be used as antibacterial agents in a lower dose than when administered systemically. These materials are typically polycationic and act through membrane disruption, making it difficult for bacteria to develop resistance. Thus, many of such polymers are active against current strains of multi-drug resistant bacteria and could be considered as a valuable method for the treatment of multi-drug resistant bacterial infection [45]. Several natural polymers, such as chitosan and gelatin, exhibit substantial antimicrobial activity and were used for wound healing and preventing bacterial infection [46]. Different strategies were developed to increase the antibacterial activity of hydrogels that can be used for wound dressings. Chitosan hydrogels can be used alone or combined with antibiotics and/or metal nanoparticles (NPs) [47]. Antimicrobial activity of chitosan hydrogels can be improved by including tertiary amino groups along the backbone [48]. Additionally, chitosan or alginate based hydrogels containing honey, which provide wound healing, reduce pain, and prevent infection of surgical wounds, are available on the market [49]. Hydrogels have been widely used in practical fields, such as pharmaceuticals, biomedical implants, food additives, regenerative medicine, artificial biostructures, diagnosis, cell immobilization and encapsulation, biosensors, barrier materials for molecular and cell separation, and for the adjustment of biological adhesion, microelectromechanical systems, and controlled drug release. Recent developments brought hydrogels in competition with many previous smart functional materials used for countless applications. The spectrum of functional monomers and macromeres keeps widening its applicability [27,50]. Hydrogels can be produced using antimicrobial polymers. This approach would limit the risks of bacterial resistance and prevent involvement of harmful items. Polysaccharides form a class of biomaterials which deserve special attention and represent the main structural component of hydrogels (Table 2).

#### 4.1.1. Microbial Sources

- Gellan gum is an anionic product extracellularly secreted by *Sphingomonas elodea* (ATCC 31461) following a microbial fermentation process [61]. It has a structure of linear polysaccharide, formed by a repeating tetrasaccharide unit made of two D-glucose, one L-rhamnose and one D-glucuronic acid (Table 2). This gum has two commercialization forms: high acyl (acetylated) gellan gum and low acyl (deacetylated) gellan gum. Both of them are capable of gelation. The difference is that the acetylated one makes elastic and translucent gels, while the deacetylated form produces gels which are rigid and, thus, more suitable for tissue engineering and regenerative medicine applications. The gelation process is conducted by a two-step mechanism [62]. The first step is a thermic process. The aqueous solution of gellan gum is heated above 80 °C for about 25 min and then cooled, driving the formation of highly ordered double helices from the linear polymers of gellan gum with randomly coiled chains. Afterwards, the cations are added and the helices are cross-linked to complete a stable hydrogel. There are several favorable characteristics of gellan gum hydrogels, including biocompatibility, similarity in structure with the inner glycosaminoglycans of the body, and mild conditions of gelation, that facilitate the incorporation of cells, making gellan gum-based hydrogels appropriate for various tissue engineering and regenerative medicine applications [51,63].
- Xanthan gum is extracellularly secreted by bacteria of the genus *Xanthomonas*, resulting from polysaccharide fermentation [64]. It is not toxic. Xanthan gum is a polysaccharide with a branched structure and is made of a repeating unit of D-glucose, D-mannose, and D-glucuronic acid, having the molar ratio of 2:2:1 (Table 2) [65]. Its harmless nature and shear characteristics make it promising for attaining an injectable scaffold for cartilage tissue repairing and for biocompatibility [66]. Xanthan gum is produced by a single-step thermic gelation process. A colloidal heterogeneous suspension, made of pockets of molecular assemblies, is constituted when, at room temperature, xanthan gum polymers are added in water. If this heterogeneous suspension is brought above 40 °C, for 3 h, annealing takes place and thus the suspension becomes homogenous. After cooling the hydrogels become robust [67].
- Dextran was the first microbial polysaccharide commercially available. It is secreted by two species of bacteria, *Leuconostoc mesenteroides* and *Streptococcus mutans*. Linear alpha-1,6 and branch alpha-1,3 glycosidic linkages between glucose monomers are at the base of its edification (Table 2). Dextran is very important in medicine, being used extensively as a volume expander and antithrombotic. The downside is that dextran cannot form hydrogels in its native state. However, composite hydrogels based on dextran were developed in order to be used in tissue regeneration [68]. Dextran is also exhibiting antimicrobial features if long alkyl tail is attached at the reducing end. More explicitly, a mixture of DMSO-MeF and NaCNBH3 with excess of dodecyl or octadecyl is mediating the reductive alkylation [69,70].

#### 4.1.2. Algal Sources

Algal polysaccharides are extracted from seaweed. The genera with the highest content of polysaccharides in the dry weight are *Ulva*, *Palmaria*, *Ascophyllum*, and *Porphyra*.

- Alginate may be present in the salts located in the cell wall of brown algae or in acid form. The composition of alginate consists of 1,4-linked alternate alpha-L-guluronic acid and beta-D-mannuronic acid residues. The chemical composition of alginates slightly differs from one algae species to another. Hydrogel preparation is mainly used in the biomedical field, in drug release or tissue regeneration. For a hydrogel to be formed, divalent cations are needed. Calcium chloride is such a cation which provides the cross-linkage. The salts of alginate also exhibit antimicrobial effects. Percival et al. (2011) [71] reported effects, including the growth inhibition of infectious agents as *Streptococcus viridans* and *Candida albicans*. Such properties can be boosted by adding alkyl groups to alginate [52].
- Carrageenans. There are only three forms of carrageenans found in nature, represented by kappa, iota, and lambda. The k-carrageenan is obtained from the alga *Kappaphycus alvarezii*, while i-carrageenan is extracted from *Euchema denticulatum*. Carrageenans vary in about 15 different structural ways. They are generally made of differently linked D-galactopyranose units. Carrageenans also include sulfate groups in their structure. Several hydrogels were developed from carrageenans. For bone tissue regeneration, a sensitive medical issue, a composite hydrogel from k-carrageenan/collagen-hydroxyapatite was developed [72]. Injectable hydrogels based on the same carrageenans are produced to be involved in tissue engineering [72]. Azizi et al. (2017) [55] fabricated a bio-nanocomposite hydrogel by incorporating biosynthesized silver nanoparticles with kappa- carrageenan. Diverse plant extracts were used for the synthesis of Ag nanoparticles. It demonstrates an excellent antimicrobial effect against *S. aureus*, methicillin-resistant *S. aureus E. coli*, and *Pseudomonas aeruginosa*.

#### 4.1.3. Animal Sources

- Polysaccharides from animal sources are also widely used in order to obtain hydrogels. From them, chitin is the one most common. The animal polysaccharides are chemically modified before being used to obtain hydrogels, the native form lacking the needed characteristics. For instance, chitin needs to be transformed to chitosan. Chitin is structured by 1–4 glycosidic bonds linking N acetyl glucosamine. The highly acetylated residues present in chitin make it rigid, and therefore not suitable. Chitin is found in the exoskeleton of insects, but it is mostly obtained from crab shells, which contain a large amount of calcium, and so need to be subjected to a demineralization process. Chitosan was accidentally obtained by Rouget in 1859 [72]. Its structure consists of two units of 2-acetamido-2-deoxy-beta-D-glucan and 2-amino-2-deoxy-beta-D-glucan. The extent of the deacetylation by which chitosan is obtained determines the hydrophilicity of the final product. Native chitosan needs to be made less hydrophilic in order to be used for drug delivery systems. Suitable chitosan hydrogels can only be obtained from modified chitosan [73]. Chitosan not only has antimicrobial properties, it is also able to involve neutrophils and macrophages in the healing of wounds, thus improving its benefits. Allan and Hadwiger (1979) [74] were the first research group, who claimed that chitosan demonstrates antagonistic behavior towards fungi. Following the report by Allan and Hadwiger (1979) [74], many studies were published which discussed fungicidal and antimicrobial characteristics [73,75,76,77]. However, the exact mechanism of antimicrobial activity remains obscure. Their antimicrobial properties can even be increased by augmenting the cationic charges along the polymer backbone. Thus, it was observed that the hydrogels of quaternized chitosan, which contain tertiary amino groups, provide a reduced risk of infection and sustain tissue repair at the same time [27]. The chitosan gels are easy to prepare. Chitosan is dissolved in acetic acid, and then a sodium hydroxide solution is added until the solution reaches 9 (pH). Then, the raw hydrogel is decanted, washed, and dialyzed [78].
- Chondroitin sulfate is another source of hydrogels belonging to the glycosaminoglycans; with the compounds exhibiting linear heteropolysaccharide chains formed of repeating units of disaccharides [79]. Chondroitin sulfate can be found widely, in many different tissues (hyaline cartilage, skin, blood vessels, etc.). Barkat et al (2019) [80] used chondroitin sulfate hydrogels packed with oxaliplatin against colorectal cancer [72].
- Hyaluronic acid is a mucopolysaccharide, also formed in living organisms, present in the synovial fluid, which functions as a lubricant. Hyaluronic acid is a linear polysaccharide made of nonsulfated glycosaminoglycan units. Hydrogels based on hyaluronic acid are obtained by cross-linking. An injectable hydrogel made of hyaluronic acid is used for drug delivery in cancer therapy [57].

#### 4.1.4. Plant Sources

The properties of these polymers are related to the plant species, growth condition and harvest age, or the season. In the plant cell walls, cellulose is the most abundant compound. The plant tissue also contains lignin and hemicellulose. Polysaccharides are also stored in the form of starch [81].

- Cellulose hydrogels can be obtained by cross-linking of cellulose in the solution [72]. As cellulose has a variety of the hydroxyl group, it can easily form networks by linking through H2 bonding. Huang et al. prepared a nanofiber hydrogel with healing capacity with dialdehyde cellulose nanocrystals and carboxymethyl chitosan [82]. Double network hydrogel was achieved by diffusion of isopropylacrylamide in cellulose hydrogels cross-linked to epichlorohydrin. Double network hydrogels were analogically obtained by changing the ratio between isopropylacrylamide to acrylamide [83]. Cellulose hydrogels exhibiting remarkable stretchability can be manufactured using the sequential cross-linking and dual network techniques [84]. Fabrication in the NaOH/urea system requires two steps. The first step includes cross-linking of cellulose by epichlorohydrin. By electron microscopy techniques, it was observed the morphology of the first network is changed, resulting in improved mechanical properties. First, the precursors are diffusing within the first network, then the polymerization is UV-light initiated and, thus, dual network hydrogels are emerging.
- Locust (Carob) bean gum represents a natural nonstarch galactomannan, it is not ionically branched and can be used in various fields based on its inner flexibility. The locust bean gum and its hydrogel-derived preparations are very popular, being widely used in food, pharmaceutical, biomedical, or cosmetic fields. Locust bean gum is also used as a carrier for drug delivery applications. Alongside the use of this popular material, novel versions were obtained by different modifications processes. Locust bean gum can be used for specific functions through its combination with several other polymers. It even responds to various stimuli, enhancing the applicability in various therapies [72].

### 4.2. Synthetic Hydrogels

In addition to natural polysaccharide polymers, there are various synthetic polymers, such as poly(acrylamide), poly(vinyl acetate), and poly(ethylene glycol). The main advantage of synthetic polymers is the ability to easily modify and combine them [85]. A common augmentation includes the addition of quaternary ammonium [86]. Meanwhile, in addition to simple functional groups, polymers can be enhanced with antimicrobial peptides (AMPs). Naturally, AMPs are encoded by all lifeforms and are considered to be a part of innate immunity [87]. AMPs electrostatically disrupt the bacterial membrane [88]. One of these AMPs represents poly-lysine, which has ability to hinder both Gram + and Gram − bacteria proliferation [89]. Similar to AMPs are amphoteric gels, with a multitude of both acidic and basic groups, such as poly(norbornene). Amphoteric gels derive their antagonistic features from electrostatic interactions [90].

## 5. Composite Antimicrobial Hydrogels

**Figure 3:**
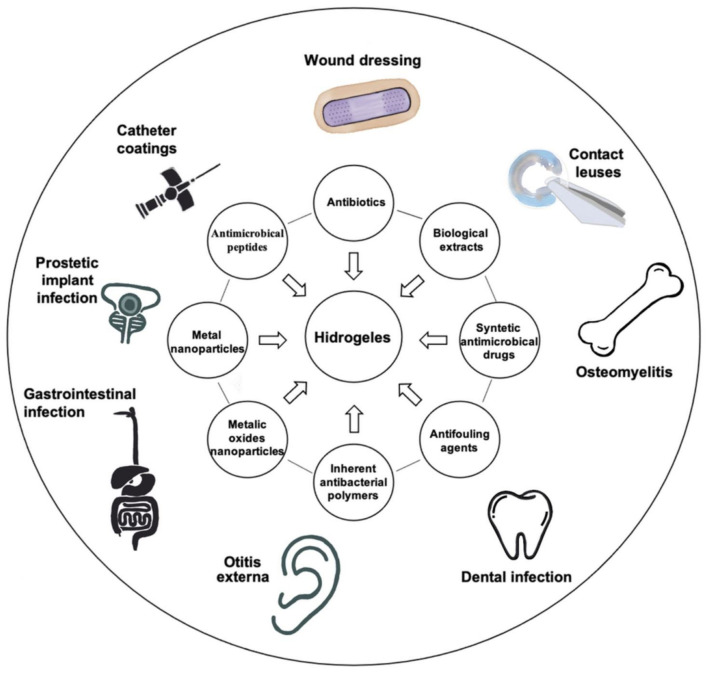
Applications of different types of antimicrobial hydrogels.

Although the high content of water in hydrogels is beneficial for wound healing processes, it also attracts bacteria, making implantable hydrogels susceptible to infections [45]. Thus, composite hydrogels could be the solution for particular treatments. Several types of hydrogels, named composite hydrogels, could covalently or physically bind different types of antimicrobial compounds, such as antibiotics, antimicrobial peptides, biological compounds, polysaccharides, and nanoparticles (NPs), to address the bacterial resistance. Composite antibacterial hydrogels have improved properties such as hydrophilicity and porosity, due to compounds added in their structure, and changes in monomer composition and the cross-linker [14]. Unfortunately, hydrogels release the antimicrobial compounds by passive diffusion of gel degradation [91], creating a dose gradient around the hydrogel matrix that may contribute to the selection of resistant bacteria; but several solutions were proposed to overcome this problem. Different types of antimicrobial hydrogels and their applications are shown in (Figure 3).

### 5.1. Chitosan Grafted Hydrogels

Chitosan’s antibacterial properties have been demonstrated by many authors. For example, chitosan interacts with the outer cell membrane in cases of *E. coli* and *Salmonella* [92], modifying its properties. Similar effects were observed in several *Candida* strains [93]. In general, changes in chitosan structure that decrease the polycationic nature of chitosan also decrease the antimicrobial activity, and increase degree off substitutions in chitosan structure enhanced antimicrobial properties [93]. Freshly-prepared alginate hydrogels embedded in the chitosan–hydrochloride solution showed higher than 99% antimicrobial activity against *E. coli* after 3 h of contact, in comparison with uncoated alginate hydrogel. After 24 h, the complete killing of bacteria was observed [94]. Grafting the chitosan with poly(acrylic acid-co-hydroxyethyl methacrylate) enhanced their activity against *S. aureus*. Coating chitosan onto alginate hydrogels improved their antimicrobial activity against *E. coli* [95]. Meanwhile, poly (Nisopropylacrylamide-co-urethane) hydrogels improved the activity against *S. aureus* and *E. coli* [96].

### 5.2. Hydrogels Containing Immobilized Antimicrobial Compounds

Incorporation of antibiotics, antimicrobial peptide, and metal nanoparticles in hydrogels or other polymers showed great promise in therapy, enhanced wound healing, prevent infection of medical devices, and had antibacterial activity against pathogens, such as *S. aureus* and *S. epidermidis* [97].

#### 5.2.1. Antibiotic-Loaded Hydrogels

As the antibiotics are the most common and effective antibacterial compounds, the development of hydrogels containing antibiotics was an alternative to overcome the bacterial resistance, by local delivering of the adequate bactericidal dose of antibiotics directly into the infected site, avoiding the systemic toxicity level [98]. Hydrogels are one of the most convenient form of local administration due to selectively release of their loaded drugs at desirable sites [99] and their biocompatibility [44]. Thus, some of the antibiotic-loaded hydrogels are summarized as follows. Ciprofloxacin can be incorporated into antibacterial hydrogels and self-assembled with a tripeptide (d-Leu-Phe-Phe). These nanostructured hydrogels have high drug loading efficiency, a prolonged release [100], nontoxicity, and antimicrobial activity against *S. aureus, E. coli* and *Klebsiella pneumoniae*. Polyacrylate hydrogels loaded with ciprofloxacin prevented the Ti implant-associated infections with MRSA, by long-term release of the antibiotic [98]. A composite hydrogel with ultraviolet-triggered ciprofloxacin release showed excellent antibacterial effects against MRSA [101]. Another type of polymer structure based on poly-vinyl alcohol and chitosan oligosaccharide was developed for antibiotic delivery applications. Ciprofloxacin HCl loaded in this polymer film showed biocompatibility and antimicrobial activity against *E. coli* and *Bacillus cereus* [102]. Local administration of gentamicin was also studied. Thus, an injectable gellan gum hydrogel with gentamicin loaded poly(lactide-co-glycolide) NPs was active against *Staphylococcus saprophyticus* without affecting the bone forming cells [103]. Another class of thermosensitive hydrogels based on chitosan-glycerophosphate incorporating nanosized hydroxyapatite/gentamicin were introduced into polymethylmethacrylate bone cement, resulting in an increased mineralization capacity and an enhanced antibacterial activity [104]. Polysaccharide gentamicin hydrogels based on pullulan derivatives also showed antibacterial activity [105]. Collagen–silica nanocomposite hydrogels loaded with gentamicin sulfate and sodium rifampicin can potentially enhance antimicrobial activity [106]. A thixotropic hydrogel with single-application and slow-releasing of gentamicin was developed for the treatment of otitis externa, and demonstrated antimicrobial activity against *Pseudomonas aeruginosa* and *Staphylococcus aureus*, the predominant bacterial strains associated with outer ear infections [107]. A charged hydrogel loaded with vancomycin was able to control the antibiotic delivery and it was used to combat the surgical site infections against MRSA [108]. The photo-cross-linked methacrylated dextran and poly(l-glutamic acid)-graft-hydroxyethyl methacrylate hydrogels also had antibacterial properties [109]. Collagen/chitosan gels incorporating norfloxacine showed enhanced properties for wound healing in rats [110] and collagen–carboxymethyl chitosan gels releasing ciprofloxacin HCL and gentamicin sulfate, and also showed promising results in a rats inducing re-epithelialization, collagen deposition, angiogenesis, and preventing wound infection [111]. A remarkable inhibitory activity against Gram + and Gram − bacteria was obtained by hydrogels cross-linked with amikacin or other aminoglycoside antibiotics, which prevent the selection of multi-drug resistant bacteria, because the antibiotic release was produced only when exposed to acid-producing bacteria [112]. Incorporation of antibiotics in the hydrogel matrix was successfully used in periprosthetic joint infections and fracture-related infections [113]. These hydrogels prevent bacterial adhesion to the implants and the formation of biofilm, increasing the treatment efficiency and reducing the time of hospitalization [19]. Several hydrogels containing antibiotics have shown great results in preclinical studies in terms of wound healing. Thus, keratin hydrogels containing ciprofloxacin effectively inhibited *S. aureus* and *P. aeruginosa* infection and enhanced skin regeneration in a porcine burn model [114]. In humans, hydrogels with prolonged release of antibiotics are not widely explored for wound healing because of the increased risk of bacterial resistance and hydrogels containing metal-based NPs are preferred [82].

#### 5.2.2. Biological Extract-Loaded Hydrogels

In the past, several plant or animal extract-loaded hydrogels were used as antimicrobial materials [115]. Thus, a seaweed extract-based hydrogel was used for wound dressing [116], allicin–chitosan was used as an antibacterial agent in foods [117] and a hydrogel with Ag-curcumin NPs was also used for their antibacterial and wound healing properties [118]. Essential oils, such as lavender, thyme, peppermint, tea tree, rosemary, cinnamon, eucalyptus, and lemongrass, encapsulated in sodium alginate hydrogels were also effective on bacteria [119]. Biological extracts from animals, such as honey and propolis, incorporated in carboxymethyl cellulose hydrogels were used for wound dressing [120]. Hydrogel contact lenses incorporated with lysozymes, derived from normal tears, exhibited remarkable antibacterial activity [121]. Some polysaccharides with inherent antibacterial activity against Gram + and Gram − bacteria, such as chitosan or carboxymethyl chitosan, were used as a matrix for the preparation of hydrogels because of their nontoxicity, biodegradability, and biocompatibility [122].

#### 5.2.3. Synthetic Antibacterial Drug-Loaded Hydrogels

Apart from semisynthetic antibiotics or biological extracts, several synthetic antibacterial drugs, such as nitroimidazoles and sulfanilamides, were used to develop antimicrobial hydrogels. Hydrogels based on polyacrylic acid and dextrin were used for the delivery of ornidazole and showed antimicrobial activity against anaerobic bacteria and amoeba from digestive system, and were nontoxic to human mesenchymal stem cells [123]. Other hydrogels based on dextrin grafted with poly(2-hydroxyethyl methacrylate) were used for drug delivery in the colon region [44]. Chitosan/gelatin/beta-glycerophosphate hydrogel containing metronidazole was tested as an injectable form for periodontal infection and it maintained the release of metronidazole in effective concentrations for *Clostridium sporogenes* [124]. Composite hydrogels based on chitosan, the acrylic acid, and N-methylene bisacrylamide could be promising antibacterial agents against a broad spectrum of Gram + and Gram − bacteria [125]. Chlorhexidine diacetate-contained poly(2-hydroxyhexyl methacrylate-co-Nisopropylacrylamide) hydrogels also showed promising thermoresponsive and antibacterial properties against *Staphylococcus epidermidis* [126]. Other studies showed that chloramine-T and sulfadiazine sodium coloaded hydrogels composed of poly-vinyl alcohol, poly-vinyl pyrrolidone, and glycerin accelerated the wound healing with an antibacterial effect [127]. Poly(N-hydroxyethyl acrylamide/salicylate hydrogels provided antibacterial activity against *E. coli* RP437 and *Staphylococcus epidermidis* and antifouling functions [128].

#### 5.2.4. Peptide Hybridized Hydrogels

**Figure 4:**
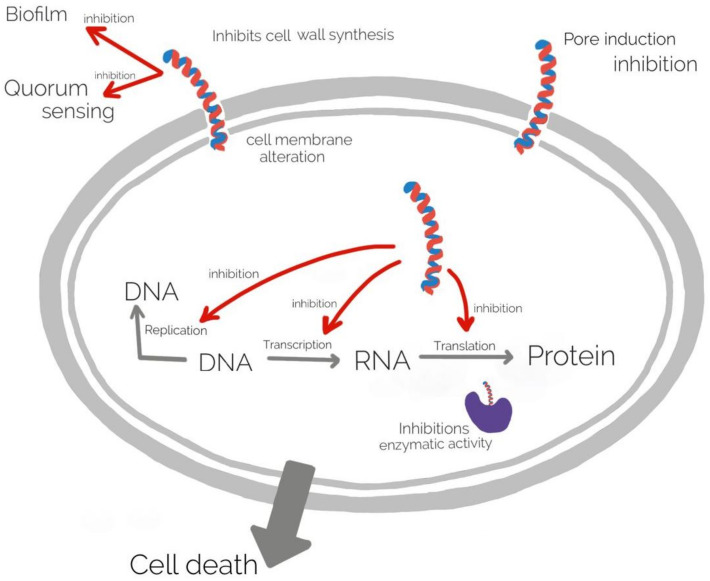
The mechanism of action of AMPs.

Antimicrobial peptides (AMPs) are a diverse group of molecules produced by plant and animal cells having a strong antimicrobial activity against Gram + and Gram − bacteria, fungi, viruses [129,130]. The mechanism of action of AMPs is complex, mainly they associate to the cell wall and membrane leading membrane alteration, and inhibition of different cellular processes as DNA replication, transcription, translation, and enzyme activity [131]. The mechanism of action of AMPs is illustrated in (Figure 4).

Covalently bounding of AMPs as Ala5-Tritrp7, ABU-CHRG01, Temporin-A, to poly (2-hydroxyethyl methacrylate) hydrogels generated composite hydrogels with improved antimicrobial activity. AMPs conjugated to polyethylene glycol maleate citrate- co-poly(ethylene glycol diacrylate) generated the biodegradable hydrogels (iFBH) with antimicrobial and wound healing properties [132]. Additionally, the hybrid antimicrobial peptide as cecropinA-thanatin incorporated in hydrogels was highly active on Gram − and Gram + bacteria [133]. Antifouling hydrogels were obtained by co-polymerization of chemically modified poloxamer 188 with poly(2-hydroxyethyl methacrylate) that were efficiently against *E. coli* adhesion on catheters [134]. Coating contact lenses with allylamine plasma polymer and polyethylene glycoldialdehyde reduced microbial contamination [135]. For therapeutic treatment of ophthalmic infections, poly(2-hydroxyethyl methacrylate) hydrogel lenses were coated with norfloxacin and the antibiotic was released over the course of several weeks [136]. Imprinted polymyxin B-loaded poly(2-hydroxyethyl methacrylate) hydrogels, for the controlled release of antimicrobial peptides, were also used in ophthalmology and were efficient against *P. aeruginosa* [137]. The antimicrobial peptide HHC10, introduced into the sodium alginate/polyethylene glycol hydrogels, displayed a strong antibacterial activity against *E. coli*, good biocompatibility, and could be used as coatings for medical devices [138]. An ultrashort peptide (naphthalene-2-ly)-acetyl-diphenylalanine-dilysine-OH (NapFFKK-OH) loaded hydrogel was formulated as a topical treatment of fungal infections relating to the skin, eyes, or as a hydrogel coating for the prevention of biomaterial related infection, being active on *Aspergillus niger, Candida glabrata, Candida albicans, Candida parapsilosis*, and *Candida dubliniensis* [139]. An amphiphilic antibacterial hydrogel with covalently bound, positively charged AMP was developed for the treatment of skin wounds, that showed prolonged (more than 48 h) antimicrobial activity against Gram +, Gram − bacteria, such as *Staphylococcus epidermidis, Staphylococcus aureus, P. aeruginosa*, methicillin-resistant *S. aureus* (MRSA), and multi-drug resistant *E. coli* [140].

#### 5.2.5. Immobilized Metal, Metal Oxide Nanoparticles

**Figure 5:**
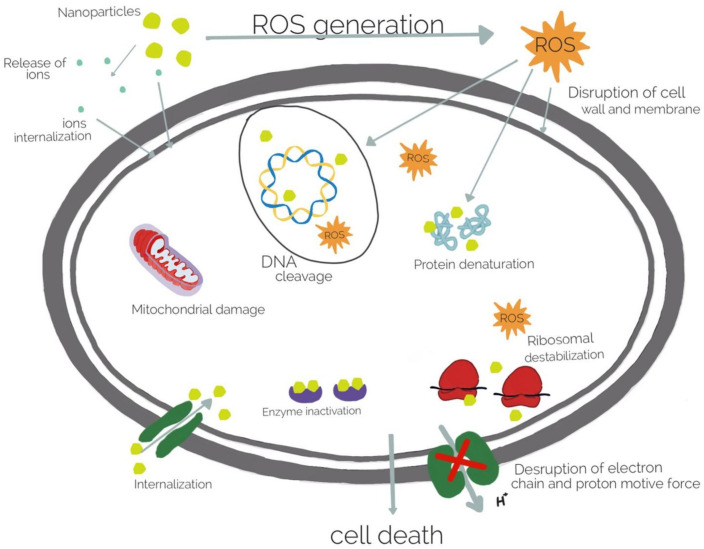
Antibacterial mechanisms of metal nanoparticles.

Although the incorporation of bactericidal agents in hydrogel is very efficient, this strategy has the disadvantage of the deposition of dead microorganisms on the surface of medical devices. Proteins mediate this process known as biofouling and can be prevented by composite repellent hydrogels incorporating metal ions and metallic oxide NPs. Commonly used metal ions include, silver (Ag), gold (Au), and copper (Cu). The most used metallic oxide metal NPs include zinc oxide (ZnO), titanium dioxide (TiO_2_), and nickel oxide (NiO). Currently, the most widely used inorganic antibacterial materials are silver nanoparticles (Ag NPs) [141] and ZnO NPs [142]. Ag NPs could be included in natural polymers or modified natural polymers, and in synthetic polymers as well [142]. The polysaccharides, such as alginate, play an important role as the natural hydrogel matrix. Sodium alginate incorporating Ag NPs showed antibacterial activity against *S. aureus* [143]. Alginate hydrogels loaded with Ag NPs have been also used in wound healing in several animal models and prevent infection [141]. The natural and biodegradable sodium alginate nanocomposite hydrogels showed a sustained release of Ag and a long-term antibacterial activity [144]. N-terminally 2-(naphthalen-6-yl)acetic acid-protected Phe-Phe-Cys peptide (Nap-FFC) hydrogel, with incorporated Ag NPs was active against both Gram + (MRSA) and Gram - bacteria (*Acinetobacter baumannii*) [145]. A thermoplastic hydrogel synthesized from multiblock PEG–POSS (POSS; poly(hedraloligosilsesquioxane)) polyurethanes incorporated with Ag NPs inhibits the biofilm formation and also showed antibacterial properties during the 14 days [146]. Acrylic acid–agar hydrogels incorporating Ag NPs showed antimicrobial activity against *E. coli* and methicillin-resistant *Staphylococcus aureus* (MRSA) under in vitro conditions [147]. Poly-vinyl alcohol, polysaccharides, and nanocrystalline cellulose were used to develop a film containing Ag NPs that could release the particles in a controlled manner for the possible use in treatment of oral or wound infection. It was demonstrated that silver cross-linked nanocrystalline cellulose was effective against *E. coli* and methicillin-resistant *Staphylococcus aureus* [148]. Polyelectrolyte hydrogels bearing amino acid residues embedded with Ag NPs showed good antibacterial activity against Gram + (*B. subtilis*) and Gram − (*E. coli*) bacteria, and higher antifungal activity against *S. cerevisiae* than native hydrogel [15]. Chitosan and chitin have inherent antibacterial and metal-binding properties, thus incorporating Ag NPs with enhanced antibacterial activity against *E. coli* and reduced toxicity [149]. Polyethylene glycol-coated Ag NPs and carboxymethyl chitosan hydrogels loaded with silver NPs were effective against both Gram + and Gram − bacteria and combined with therapeutics promoted wound healing [150]. Hybrid hydrogel lenses, composed of quaternized chitosan, Ag NPs, and graphene oxide displayed antimicrobial properties, and cytocompatibility [82]. An alginate/gelatine hydrogel loaded with Ag NPs improved wound healing in vivo in Wistar rats, was non-toxic against fibroblast and showed antibacterial activity against *Pseudomonas aeruginosa* and *Staphylococcus aureus* [151]. Gold nanoparticle (Au NPs) could also be used to design different polymer structures with biocompatibility and antimicrobial activity, but compared to Ag NPs, Au NPs are insufficiently studied [142]. Nevertheless, Au NPs loaded in gelatin hydrogel cross-linked with genipin showed antimicrobial activity when Au NPs release is triggered by thermal stimuli [63]. The Au NPs attach to cell membranes, leading to the leakage of bacterial contents or penetrate the outer membrane and peptidoglycan followed by cell death. Combining the Au NPs with antibiotics or antifouling compounds, the bacterial resistance could be reduced [128]. The hydrogel containing carboxyl-modified Au NPs absorbed onto the outer surfaces of cationic liposomes showed skin biocompatibility in mice and antibacterial activity against *S. aureus* [152]. In order to increase the antibacterial properties, bimetallic (Ag and Au) hydrogel nanocomposites were prepared. The nanoparticles obtained by green technology with mint leaf extract showed antibacterial activity against *Bacillus* and *E. coli* [153]. Other metal NPs could be used to design antimicrobial hydrogels, such as cobalt-exchanged natural zeolite/poly(vinyl alcohol) hydrogels that showed antibacterial activity against *E. coli* and *S. aureus* [154,155]. Sodium alginate complexed with Cu NPs were bactericidal effective against *E. coli* and MRSA [156]. Generally, metallic nanoparticles destroy the bacteria by attaching to cell membranes followed by its disintegration, leakage of bacterial contents and inhibition of protein synthesis (Figure 5). Moreover, the toxicity of metallic nanostructures is high even in low concentration of nanoparticles. Thus, further studies are required to investigate properties of the metallic nanoparticles in association with antibacterial properties [133]. Zinc oxide NPs alone or in combination with Ag NPs included in hydrogels have also shown antibacterial effect in a rat model for wound healing [157]. The reduced biocompatibility of zinc oxide and silver have limited their applications and more long-term studies are needed to evaluate their potential adverse effects [158,159]. Nevertheless, several topical silver-containing hydrogels which have a broad spectrum of pathogenic bacteria and fungi are available for patients, such as ReliaMed, Acticoat, Gentell Silver hydrogel, Silvermed, Silver-Sept, SilvaSorb, silvergenesis colloidal Solver hydrogel, and DermaSyn/Ag [160]. Despite their high applicability, nanoparticle-based hydrogels have some limitations. Due to the particular structure of the cell walls of Gram + bacteria, they are less effective in such bacteria [161]. Furthermore, nanoparticles are physically and chemically unstable, which also limits their uses. Moreover, the dead bacteria are deposited in this hydrogels, limiting their antifouling properties. To overcome this problem, salicylate anions or carboxylate ions were released in zwitterionic hydrogel to maintain the gel antifouling properties [162]. Metallic oxide NPs-loaded hydrogels show also good antimicrobial activity. The action of metallic oxide NPs differs from metal NPs, the main mechanism is photocatalysis under UV irradiation of sunlight, hydroxyl and oxygen radicals are produced, that oxidize the organic compounds from microorganisms that kill the bacteria [126]. ZnO is one of the most popular metallic oxide used in different composite hydrogels for their antibacterial activity [163]. It is also non-toxic, thus it can be used in cosmetics and for wound healing and collagen deposition [164]. It was shown that such hydrogels are effective against Gram + and Gram − bacteria and against resistant bacterial spores [165]. Sodium alginate hydrogel loaded with ZnO NPs showed very good antimicrobial activity against *E. coli, S. aureus, Candida albicans*, methicillin-resistant *S. aureus* and was nontoxic on human dermal fibroblasts at low concentrations of ZnO [166]. Alternatively, ZnO NPs incorporated in poly(N-isopropylacrylamide) gel were used as coating for biomedical device, showing antimicrobial activity against *E. coli* and no cytotoxicity toward the mammalian cell line (3T3) over one week [29]. Carboxymethyl cellulose nanocomposite hydrogels incorporated with CuO NPs showed excellent antibacterial effects against Gram + and Gram − bacteria [167] and TiO_2_ NP-loaded chitosan–pectin composite hydrogel generated wound dressings with photoactive property, excellent biocompatibility, and good antibacterial activity [168].

#### 5.2.6. Carbon Material-Loaded Hydrogels

Several carbon nanotube hydrogels showed antimicrobial activity against *S. aureus, E. coli,* and *Candida tropicalis* [169], and graphene oxide also exhibited strong antibacterial activity against Gram + and Gram − bacteria [170]. Other authors showed that an Ag/reduced graphene oxide hydrogel exhibited good antibacterial activity against *E. coli* and *S. aureus* and has excellent biocompatibility [171].

## 6. Hydrogels as Carriers of Antimicrobial Agents

Hydrogels have been designed as a carrier for antimicrobial agents as antibiotics and other synthetic antimicrobial substances, metal/metal-oxide NPS, AMPs, and biological extracts [14], to overcome the problems generated by standard drug administration due to high dosage, repeated administration and toxicity [52]. Several strategies were developed in order to increase hydrogel antimicrobial activity, to control antimicrobial release, and to reduce the toxicity of biocidal agents as follows: (a) physical incorporation of NPs in hydrogels, (b) integration of enzyme cleavage sites into hydrogels, (c) optimization of hydrogel properties, and (d) development of bacteria responsive hydrogels [14].

Generally speaking, the mechanism of classical antimicrobial agents, such as antibiotics, are based on biochemical interactions. Despite multiple structural classifications of antimicrobial compounds, most of them hinder cell growth by: (1) cell wall destruction, (2) inhibition of protein synthesis, and (3) nucleic acid metabolism [172]. For example, a cell wall can be disrupted by vanxomycin. This glycopeptide binds to peptide side of the peptidoglycan precursor, reducing cell wall thickness. The other antibiotics affect the protein synthesis backbone. Specifically, tetracyclines and macrolides inhibit 30S and 50S ribosomal subunits. Meanwhile, fluoroquinolones hinder transcription by DNA gyrase inhibition [172,173,174].

As a whole, antibiotics represent an efficient and sophisticated method of cell growth inhibition. In comparison to antibiotics, inherent antimicrobial polymers represent a more diffused way of inhibition. Generally, polymers such as chitosan increase bacterial outer membrane permeability, leading to the release of cellular components [92,175]. Thus, inherent microbial polymers are not quite as efficient as antibiotics and must be modified either with Ag or alkylation [52].

### 6.1. Physical Incorporation of NPs in Hydrogels

Controlled antimicrobial release was obtained by different strategies, such as: physical incorporation of metal NPs in hydrogels as cellulose–polymer–Ag nanocomposite fibers [176], or Au NPs incorporated in composite hydrogels [144], NP-stabilized liposomes [152] and antibiotic-loaded NPs [177]. The release efficiency of gold NP-stabilized liposomes containing antimicrobial compounds was obtained by increasing of the cross-linker concentration and released of lyposomes from the hydrogel was pH dependent. This composite hydrogel had antimicrobial activity on *S. aureus* at pH 4.5 [152]. Improved adhesive strength in wet environments was obtained by combination of ciprofloxacin-loaded poly(lactid-co-glycolide) NPs with dopamine methacrylamide. Dopamine increased by 92%, with the retention of ciprofloxacin-loaded NPs in wet conditions, and by 40% with the gradual release of ciprofloxacin within 12 h compared with control, which was 94% within 12 h [177]. Injectable hydrogel consisted of gentamicin-loaded poly(lactid-co-glycolide) NPs incorporated into a gellan gum hydrogel showed improved antimicrobial activity against *Staphylococcus saprophyticus* than free gentamicin [103].

### 6.2. Integration of Enzyme Cleavage Sites into Hydrogels

Integration of enzyme cleavage sites into hydrogels was performed for controlled drug delivery, mediated by enzymatic degradation of hydrogels. Thus, a composite chitosan-based hydrogel with incorporated cefuroxime was used for wound healing, the antibiotics being released in the presence of esterases, with abundant enzymes at wound sites [178]. Another combination of antibacterial alginate-based hydrogels containing levofloxacin with alginate lyase was used for controlled antibiotic release mediated by pH [179]. Other compounds having antimicrobial activity were also used for wound healing. For example, immobilized cellobiose dehydrogenase immobilized in succinyl chitosan/carboxymethyl cellulose releases hydrogen peroxide by enzymatic gel degradation [180]. The same hydrogel matrix with incorporated cellulase was used for hydrogen peroxide release, that ensured the antimicrobial activity of the gel against *E. coli* and *S. aureus*, whereas free hydrogel has no antimicrobial activity [180].

### 6.3. Optimization of Hydrogel Properties

Hydrogel antimicrobial properties can be modulated by several parameters, including overall charge [108], polymerization method [181], monomer composition [182], cross-linker concentration [152], and antimicrobial concentration [112]. Extended release of vancomycin (during 4 days) was obtained by incorporation of the positively charged vancomycin within oligo (poly(ethylene glycol) fumarate) sodium methacrylate hydrogel which is negatively charged, ensuring the efficient loading of vancomycin without affecting its potency [108]. On the other hand, oxidized polysaccharide hydrogels (dextran, carboxymethyl cellulose, alginate, and chondroitin sulfate) using aminoglycosides as cross-linkers were more efficient on different bacteria than a calcium cross-linked alginate hydrogel encapsulating aminoglycoside [112]. Moreover, hydrogels with varying amikacin concentrations were more effective on *E. coli, S. epidermidis, S. aureus, P. aeruginosa* than commercially available hydrogels (e.g., Nano-Ag, Achromycin gel) [112]. Biodegradable hydrogels based on poly (D,L-lactic acid) encapsulating gentamicin or teicoplanin, were used for progressive release of antibiotic over a period of 96 h [183], that is useful for reduction in bacterial adhesion and viability on medical devices as implants, catheters, and to reduce post-surgical infections [184,185]. In order to reduce coating thickness of titanium implants and controlled released of antibiotic, the electrosynthesis of poly(2-hydroxyethyl methacrylate) or poly(ethylene glycoldiacrylate)-co-poly(acrylic acid) loaded with ciprofloxacin [98] or Ag NPs was used [186], having antimicrobial activity against *S. aureus, P. aeruginosa*, and *E. coli*.

### 6.4. Development of Bacteria Responsive Hydrogels

Hydrogels can be developed to respond to different stimuli as temperature [187], pH [188], light [159], electricity [188], and bacteria [177,189]. Smart antibacterial hydrogels have been developed to respond to biological stimuli related to the presence of bacteria, such as changes in pH or bacterial enzyme secretion [112,190]. Hydrogels responding to a bacterial stimulus could be obtained by the use of proteases and virulence factors produced during an infection. A gelatin methacryloy hydrogel embedded with 10,12-tricosadiynoic acid vesicles containing antimicrobials was designed to recognize the pathogenic bacteria. The specific release of antimicrobials is triggered by pore inducing toxins of *P. aeruginosa* or *S. aureus*, while nonpathogenic *E. coli* did not produce toxins and the vesicles remain intact [191]. Nanogels coated with red blood cell membranes were designed to target MRSA [177] and hyaluronic acid hydrogels were designed to release Fe${}^{3+}$ that in combination with H${}_{2}$O${}_{2}$ forms hydroxyl radicals leading to bacterial cell death [189]. A complex hydrogel based bifunctional coating for urinary catheters that can both detect and inhibit bacteria by modification of the surrounding pH due to infection was developed. *Proteus mirabilis* hydrolyzes urea, that is followed by the increases of the urinary pH. The polydiacetylene vesicles detect this changes by visible color transition, from blue in acidic media (pH < 7), to purple and red in alkaline media (pH 7–8.8 and pH > 8.8, ensuring the bacterial detection and the releasing of ciprofloxacin at pH 7 [191]. Another way to create a pH-sensitive hydrogel is to incorporate Schiff bases. Schiff bases contain C=N double bond, which is unstable at acidic pH and allow gel degradation by changing the pH. If the hydrogel is loaded with amoxicillin, the antibiotic release could be obtained by immersion in a phosphate buffer saline dependent of the pH values [112]. Dextran aldehyde hydrogel complexed with cationic dendrimers (amine-terminated generation 5 polyamido-amine and Ag NPs in acidic environment releases the dedrimers and Ag NPs two times higher than in a neutral environment after 24 h and have a synergic effect in the treatment of bacterial infections [192].

## 7. Delivery Systems for Gene Editing Tools for Curing the Bacterial Resistance

**Figure 6:**
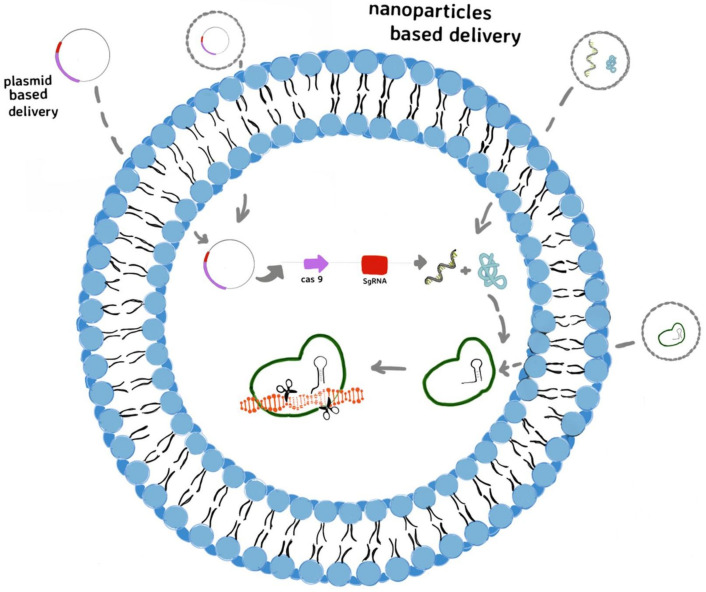
Non-viral delivery for CRISPR-Cas9 system to edit genes involved in bacterial resistance to anibiotics: the plasmid form of CRISPR-Cas9 system can be transferred into cells as plasmid or as plasmid integrated in lyposome and then transcribed into Cas9 mRNA and sgRNA. After translation, the Cas9 protein forms a ribonucleoprotein (RNP) complex with sgRNA that edits the target genes, directed by sgRNA; delivery the CRISPR-Cas9 system in its mRNA form, included in liposome; delivery the CRISPR-Cas9 in its protein form as RNP complex included in liposome.

The Clustered Regularly Interspaced Short Palindromic Repeats (CRISPR) and CRISPR-associated protein (Cas) as immune system of bacteria can detect and degrade foreign genetic material from viruses and plasmids [193,194]. It is known that the CRISPR-Cas9 system is the most widely applied in gene editing [195], the precise designing of guide RNAs targeting specific sequences allowed the employment of this system as promising tools against the multi-drug resistance prevalence as well [196]. The CRISPR-Cas 9 system could be addressed to treat multi-drug resistance in two different strategies: (i) targetting specific resistant bacteria possessing specific sequences (constitutive genes) from bacterial communities; (ii) targetting the antibiotic resistance genes, and resensitizing the bacteria to antibiotics [197]. Despite the efficiency and specificity of CRISPR-Cas9 system in targeting the multi-drug resistant pathogens or their resistance genes, its delivery has become the real challenge and major limitation for therapeutic applications [198]. Mostly, the virus-based delivery systems have been studied, due to their ability to infect bacteria [199,200]. Electrotransfer of CRISPR system (pCasCure—eliminate endemic plasmid types that confer resistance to carbapenems) to various CRE isolates—including *K. pneumoniae*, *E. coli* and *E. hormaechei*—in order to perform the deletion of KPC, NDM, and OXA-48 carbapenemases was also reported [201]. In the last years, non-viral gene delivery systems, such as polymers and nanoparticles, have been developed [202] due to their simplicity and various possibilities to design the appropriate delivery system (Figure 6).

**Figure 7:**
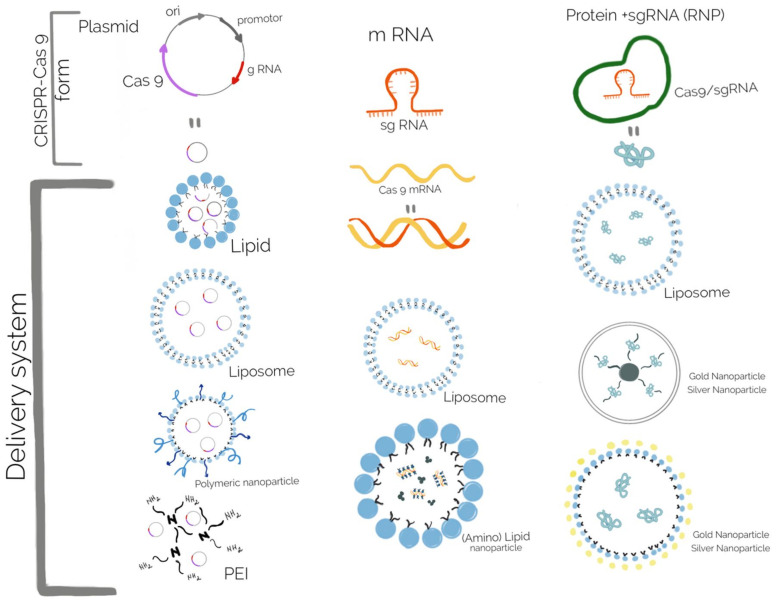
Biomaterials used for the delivery of different forms of CRISPR-Cas9 system: the DNA form delivered in lipid, liposome, polymeric nanoparticle, and PEI; the mRNA form delivered in liposome and (amino) lipid nanoparticle; the protein form delivered in liposome and different types of gold and silver nanoparticles.

Lipid and polymeric nanoparticles have more potential for future use, due to their advantages, such as low toxicity and immunogenicity, and multiple tailoring ways [203]. Lipid nanoparticles, such as liposome-templated hydrogel nanoparticles are one of the most extensively explored nanoparticle systems for CRISPR-Cas9 delivery, especially for gene therapy in cancer [204], but in bacteria the most used delivery systems are based on polymeric NPs and gold nanoparticles (Au NPs) [205]. The CRISPR-Cas9 system could be released in its DNA, mRNA, or protein form, thus different biomaterials for delivering the system were rapidly developed (Figure 7). For curing the bacterial resistance and virulence, the DNA form of the CRISPR-Cas9 system is used, but several aspects regarding the proper biomaterials developed for all delivery forms are discussed here.

Delivery of the CRISPR-Cas9 system in a DNA form consists of a plasmid containing the sequences encoding the Cas9 nuclease, a promoter to begin transcription, and sgRNA. Carrier particles for these plasmid vectors should be employed because they cannot pass through the cell membrane. Moreover this carrier particle protects the plasmids from enzymatic degradation [206]. Several cationic lipids, such as Lipofectamine, and polymer-based delivery systems, such as Turbofect (cationic polymer), are commercially available through transfection reagents [207]. Increased transfection efficiency, both in vitro and in vivo, when compared with commercial transfection reagents, was obtained by encapsulation with a cationic lipid mixture of 1,2-Dioleoyl-3-trimethylammonium propane and 1,2-Dioleoyl-sn-glycero-3-phosphoethanolaminand cholesterol [177]. Delivery of the CRISPR-Cas9 system in its mRNA form offers several advantages over plasmid DNA, mostly avoiding the need to access the bacterial chromosome, leading to quick and transient Cas9 expression, but at the same time has the disadvantage of its instability and susceptibility to degradation. Therefore, many strategies consist of coating the mRNA with positively charged groups to improve cell entry. The most of the vectors are cationic liposomes, but other vectors include functionalized (amino) lipid nanoparticles [20]. Delivering Cas9 in its protein form allows the quickest action, because the Cas9 nuclease can directly form the ribonucleoprotein (RNP) complex with the sgRNA and reach the target genes. Unlike mRNA and plasmid DNA, the Cas9 protein is positively charged, that allows interactions with the cell membrane, but, at the same time, its inability to interact with cationic lipids or polymers would leave the protein susceptible to degradation by proteases [208]. Thus, several strategies were developed to overcome this issue: to modify the Cas9 protein with a negatively charged molecule (anionic peptide or protein) or to complex Cas9 protein with an sgRNA to form a negative charged structure [209]. A complex delivery system was developed based on branched polyethylenimine (PEI) that ensured the interaction with free thiol groups found on the cysteine residues of Cas9. The cationic PEI-Cas9 was then complexed with sgRNA targeting mecA, from methicillin-resistant *Staphylococcus aureus*(MRSA). This nanocomplex shown greater bacterial uptake and decreased number of colony forming units, decreased growth rate in comparison with a simple mixture of unmodified Cas9 RNP with PEI or Lipofectamine [210]. This system may be a significant achievement in the delivery of antimicrobials in Gram + bacteria, due to their particular structure of the cell wall. As drug carriers in different types of tissue, inorganic nanoparticles, such as gold NPs, have also been used for the delivery of CRISPR-Cas9, but usually not in bacterial cells [211]. Several nanoparticles delivering the CRISPR-Cas9 system were reviewed by Deng et al., 2019, Wan et al., 2019 and Kim et al., 2020 [138,212,213] most of them used for gene editing of eucariote cells.

## 8. Challenges of Development and Uses of Antibacterial Hydrogels

Despite their numerous advantages, as described in previous sections, antimicrobial hydrogels have many issues of their own, which is why many of them have shown promising in vitro results but lack convincing preclinical investigations, and just a limited number of such compound are readily available on the market or for therapy. Naturally derived biomaterials have the advantage of the high biocompatibility and bioactivity, but in the same time, have the disadvantage of difficult processing and batch variability. Even though new and performant materials were developed, the immunity related complications are not solved, for example, the immunogenicity of the biomaterials themselves and the immune response associated with their administration. The most discussed issue is PEGylation of nanoparticle surface in order to protect against innate immune response, to reduce protein adsorbtion and increase blood circulation, that was followed by production of antibodies reactive to PEG, reducing the benefits of PEGylation [214,215]. Thus, more studies are needed to overcome this issue. Moreover, several lipids also have the potential to elicit innate and/or adaptive immune responses [216]. In other words, these materials should be cell-friendly and, at the same time, very effective on the most virulent and resistant bacteria. Synthetic materials have the main advantage of the huge tunability for tailored nanoparticles, but, unfortunately, they lack bioactive motifs for influencing the cellular processes [20]. Hydrogels carrying antibiotics are associated with the risk of selection of new resistant bacteria, thus the hydrogels based on nanoparticles and antimicrobial peptides could be a promising solution. Among commercially viable antimicrobial hydrogels, there are a notable number of silver NP-based and chitosan-based hydrogels because most of the metal or metal oxide nanoparticle are physical and chemical unstable and have reduced biocompatibility [161]. Polymeric NPs, conjugated or not with cell-penetrating peptides on the surface for quick cellular uptake and/or nuclear localization signal peptides for delivery of gene editing tools inside cells have low immunogenicity and high biocompatibility, but more studies are needed in this regard [21].

## 9. Conclusions and Perspectives

Numerous types of biomaterials, such as hydrogels and composite polymers, have demonstrated their versatility and tunability, making them a very attractive option for limiting the antimicrobial resistance, mostly in pathogen bacteria, but not exclusively. Resistant bacteria have become a global health threat, as infections are becoming more difficult to treat, thus many strategies to overcome this problem were developed and the antimicrobial hydrogels proved their numerous advantages as antimicrobials, or as carriers of antimicrobial compounds and genetic tools for curing the antimicrobial resistance of bacteria. Naturally derived biomaterials possess high biocompatibility and bioactivity, being preferred for wound treatment and preventing bacterial colonization of the medical devices, such as implants, catheters, and contact lenses. Chitosan is the most widely explored polymer for such applications, being a natural polysaccharide that can be easily modified to improve its properties and biocompatibility. Moreover, several compounds, such as antibiotics, metal NPs and AMPs, and antibiofouling compounds, could be included in natural biopolymers or in composite hydrogels to improve their antimicrobial activity. Alternatively, hydrogel backbones can be modified to obtain a prolonged or smart stimuli responsive drug release. Nevertheless, more studies are required regarding the development of composite antimicrobial hydrogels that could prevent the antimicrobial resistance due to antibiotic use and cytotoxicity of metal nanoparticles. Antifouling hydrogels could also be a promising solution against the deposition of biological molecules or bacterial cells. Moreover, for therapeutic success, the antimicrobial hydrogels should be biocompatible and must achieve bactericidal drug levels and sustained antimicrobial action. Therefore, the constant development of new strategies of designing hydrogels coupled with antimicrobial therapies will enable the potential solutions to prevent antimicrobial resistance and the successful treatment of infectious diseases.

## Figures and Tables

**Table 1:** Various types of physical (1) and chemical (2) hydrogels (cross-linking hydrogels).

Type	Crosslink	Hydrogels (Polymers)	Applications	Ref.
1	Freeze-thawing	Polyvinyl alcohol, Polyvinyl alcohol/gelatin, etc.	Therapeutic	[29]
1	Hydrogen bonding	Hyaluronic acid	Drug delivery; regenerative medicine	[30]
1	Ionic interaction	Chitosan	Antigen delivery	[31]
1	Heat-induced aggregation	Alginate capsules	Cartilage tissue	[32]
1	Stereocomplex formation	Dextran, poly lactic acid	Drug delivery	[33,34]
2	Chemical cross-linking	Polyethylene glycol	Biomedical	[35]
2	Polymerization	Polyethylene glycol methyl ether metacrylate	Antifouling	[36]
2	Enzymatic reaction	Chitosan	Packaging and wound dressing	[37]
2	Radiation	Poly oligo-propylene glycol methacrylate	Biomedical	[38]
2	Chemical grafting	Poly epsilon-caprolactone	Tissue engineering; cell viability	[39]
2	Condensation reaction	Nanocellulose crystals	Cell adhesion; viability	[40]

**Table 2:** Natural polymers from inherently antimicrobial hydrogels (1 = microbial source, 2 = algal source, 3 = animal source, 4 = plant source).

Type	Polymers	Source	Structure	Ref.
1	Dextran	*Streptococcus mutans*, *Leuconostoc mesenteroides*, etc.	Consist of (1,6) glycosidic linkages between D-glucose monomers, with branches from (1,3) linkages	[43]
1	Xanthan gum	*Xanthomonas campestris*	Composed of a pentasaccharide repeating unit, consisting of D-glucose, D-mannose and D-glucuronic acid the molar ratio of 2:2:1.	[36,51]
1	Gellan gum	*Sphingomonas elodea*	Composed of a tetrasaccharide repeating unit, consisting of two residues of D-glucose, one residue of L-rhamnose and one residue of D-glucuronic acid.	[51]
2	Alginate	Brown algae (*Phaeophyceae*)	Composed of beta-D-mannuronic acid and L-gluronic acid. Its reticulation can also occur by divalent cations (Ca${}^{2+}$, Fe${}^{2+}$, Ba${}^{2+}$)	[52,53]
2	Agarose	Red algae, (*Rhodophycae*—*Gelidium*, *Gracilariae*)	It is a linear polymer made up of the repeating unit of agarobiose, which is a disaccharide made up of D-galactose and 3,6-anhydro-L-galactopyranose	[54]
2	Carrageenan	Red algae, (*Rhodophycae*—*Gelidium*, *Gracilariae*)	The presence of L-3,6-anhydro-L-galactopyranose rather than D-3,6-anhydro-L-galactopyranose units and the lack of sulfate groups	[55]
3	Chitosan	Crustacean skeleton	It is a polysaccharides from chitin and it is composed by the repetition of N-glucosamine units.	[36]
3	Hyaluronic acid	Synovial fluid; articular cartilage.	Composed of D-glucuronic acid and N-acetyl-D-glucosamine, linked via alternating (1-4) and (1-3) glycosidic bonds	[56]
3	Chondroitin sulfate	Extracts of cartilaginous cow and pig tissues; shark, fish, and bird cartilage.	It is a sulfated glycosami-noglycan composed of a chain of alternating sugars (N-acetylgalactosamine and glucuronic acid)	[57]
4	Cellulose	Cell wall of green plants	It is an organic compound, a polysaccharide consisting of a linear chain of several hundred to many thousands of (1-4) linked D-glucose units.	[58]
4	Guar gum	Guar bean (*Cyamopsis tetragonoloba*)	Composed of the sugars galactose and mannose.	[59]
4	Locust bean gum	Seeds of the carob tree	A natural nonstarchgalactomannan	[60]

## Data Availability

Not applicable.
